# Temporal Preparation, Impulsivity and Short-Term Memory in Depression

**DOI:** 10.3389/fnbeh.2019.00258

**Published:** 2019-11-22

**Authors:** Tzu-Yu Hsu, Hsin-Chien Lee, Timothy Joseph Lane, Marcus Missal

**Affiliations:** ^1^Graduate Institute of Mind, Brain and Consciousness, College of Humanities and Social Sciences, Taipei Medical University, Taipei, Taiwan; ^2^Brain and Consciousness Research Center, Taipei Medical University-Shuang Ho Hospital, New Taipei City, Taiwan; ^3^Department of Psychiatry, School of Medicine, College of Medicine, Taipei Medical University, Taipei, Taiwan; ^4^Department of Psychiatry, Taipei Medical University Hospital, New Taipei City, Taiwan; ^5^Research Center of Sleep Medicine, College of Medicine, Taipei Medical University, Taipei, Taiwan; ^6^Graduate Institute of Medical Humanities, College of Humanities and Social Sciences, Taipei Medical University, Taipei, Taiwan; ^7^Division of System and Cognition, Institute of Neurosciences (IONS), Université catholique de Louvain (UCLouvain), Brussels, Belgium

**Keywords:** memory, depression, temporal cognition, eye movement, impulsivity

## Abstract

Patient suffering of major depressive disorder (MDD) often complain that subjective time seems to “drag” with respect to physical time. This may point toward a generalized dysfunction of temporal processing in MDD. In the present study, we investigated temporal preparation in MDD. “Temporal preparation” refers to an increased readiness to act before an expected event; consequently, reaction time should be reduced. MDD patients and age-matched controls were required to make a saccadic eye movement between a central and an eccentric visual target after a variable duration preparatory period. We found that MDD patients produced a larger number of premature saccades, saccades initiated prior to the appearance of the expected stimulus. These saccades were not temporally controlled; instead, they seemed to reflect reduced inhibitory control causing oculomotor impulsivity. In contrast, the latency of visually guided saccades was strongly influenced by temporal preparation in controls; significantly less so, in MDD patients. This observed reduced temporal preparation in MDD was associated with a faster decay of short-term temporal memory. Moreover, in patients producing a lot of premature responses, temporal preparation to early imperative stimuli was increased. In conclusion, reduced temporal preparation and short-term temporal memory in the oculomotor domain supports the hypothesis that temporal processing was altered in MDD patients. Moreover, oculomotor impulsivity interacted with temporal preparation. These observed deficits could reflect other underlying aspects of abnormal time experience in MDD.

## Introduction

Major depressive disorder (MDD) is often associated with an altered awareness of the passage time. Indeed, MDD patients often complain that subjective time is going by at a reduced pace compared with physical time (Gallagher, 2012; Msetfi et al., 2012; Ratcliffe, 2012; Droit-Volet, 2013; Thönes and Oberfeld, 2015; Davalos et al., 2018; Vogel et al., 2018). This perturbed time awareness has led to a systematic investigation of time “perception” using quantitative methods requiring an *explicit* judgment about durations. An explicit judgment about duration is the outcome of experimental tasks requiring comparison of time intervals, production or reproduction of a standard duration or verbal estimation (see Vatakis et al., 2018). This approach has led to conflicting results and the precise influence of depression on time perception remains elusive (see review in Oberfeld et al., 2014). However, temporal cognition is not limited to *explicit* temporal judgments. *Implicit* timing refers to the capacity to time actions based on temporal regularities in the environment (Coull and Nobre, 2008; Coull and Droit-Volet, 2018). It emerges in non-temporal tasks where temporal information is, nevertheless, essential to achieve optimal performance, as when making a saccade to a visual target. This implicit influence of elapsed time on movement preparation is often referred to as “temporal preparation” and is still poorly understood in depression (see Bonin-Guillaume et al., 2004).

Temporal preparation is studied, classically, by using a warning stimulus (S_1_) that predicts the occurrence of an imperative stimulus (S_2_; Woodrow, 1914). The period between S_1_ offset and S_2_ onset is referred to as the foreperiod (“FP”; Niemi, 1981; Niemi and Näätänen, 1981). Temporal preparation builds-up while waiting during the FP and causes a shorter reaction time after S_2_ appearance. Foreperiod duration could either remain constant, making the timing of S_2_ entirely predictable, or FP duration could vary randomly between different values drawn from a given probability distribution. If FP duration is randomly drawn from a uniform probability distribution, the latency of the motor response to S_2_ decreases with elapsed time. This “foreperiod effect” is the behavioral measure of temporal preparation. Temporal preparation could be explained by hypothesizing that subjects estimate the hazard rate of the target defined as the probability that S_2_ will occur given that it has not occurred yet. As time elapses during the FP, the hazard rate of the S_2_ increases and sensorimotor systems could use that information to reduce reaction time (Trillenberg et al., 2000; Janssen and Shadlen, 2005; Nobre et al., 2007). In addition, temporal preparation could also be modulated by the previous FP experienced by the subject (Alegria and Delhaye-Rembaux, 1975; Los and Van den Heuvel, 2001; Los et al., 2014, 2017). For instance, reaction time to S_2_ appearance during the current FP will tend to be shorter, if the previous FP was shorter. Therefore, short-term temporal memory (i.e., sequence effects) plays a crucial role in temporal preparation (Los et al., 2017). Accordingly, it has been shown that the FP effect on saccadic eye movements could be accounted for by the remaining trace of previous FP duration (Ameqrane et al., 2014). This influence of short-term memory on the RT-FP function could be altered given the known impact of depression on memory (Burt et al., 1995). Therefore, altered temporal cognition in depression could be mainly due to a deficit of temporal memory.

Another factor that deeply influences response preparation in general is inhibitory control (Greenhouse et al., 2015; Lebon et al., 2016; Duque et al., 2017). More precisely, in order to prevent premature responses (i.e., responses before the onset of S_2_), inhibition is necessary to reduce the increasing tendency to initiate a motor response as time elapses (Burle et al., 2010; Correa et al., 2010). Inhibitory control is not only important for current FP, but also associated with short-term temporal memory. It has been suggested that when the preceding FP is longer than the current FP, inhibition induced during the preceding FP could cause a longer RT during the current FP. This indicates that inhibition could modulate the influence of short-term memory during the FP (Los, 2013). Therefore, it is plausible that the FP effect could be altered because of a dysfunctional inhibitory control. Inhibitory dysfunction is one aspect of impulsivity (Evenden, 1999). Impulsivity could be defined as the tendency to act without forethought and is commonly considered as one aspect of personality trait. To evaluate the magnitude of impulsivity, the Barratt Impulsiveness Scale (BIS-11; Patton et al., 1995) is often adopted. Studies have shown that impulsivity was exacerbated in neurologic (e.g., Parkinson’s disease, Voon et al., 2011; Nombela et al., 2014), psychiatric (e.g., ADHD, Barkley et al., 2001; Smith et al., 2002) and affective disorders (e.g., MDD; see review in Evenden, 1999; Dalley and Robbins, 2017). There is a strong association between depression and impulsivity, suggesting that impulsivity could potentially lead to an increased risk of suicide (Corruble et al., 2003). Therefore, increased impulsivity in depression could cause more premature motor responses, altered temporal preparation and a different RT-FP function.

The aim of the present study was two-folds. Firstly, we examined whether temporal processing was altered in depressive patients by using a saccadic version of temporal preparation. Precise and accurate control of the timing of eye movements is essential to “catch” with the fovea the image of visual objects (Badler and Heinen, 2006; de Hemptinne et al., 2007; Barnes, 2008; Collins and Barnes, 2009). The same scenario also applies to temporal preparation. When both S_1_ and S_2_ are visual targets, temporal preparation plays a major role in oculomotor control (Oswal et al., 2007; Ameqrane et al., 2014; Degos et al., 2018). The saccadic system is kept under constant top-down inhibitory control in order to avoid unwanted eye movements that could blur the image of a visual object of interest on the retina during fixation periods (Rucker et al., 2011). The saccadic system therefore could be particularly sensitive to a lack of inhibitory control during temporal preparation and premature saccades and could be a valuable indicator of impulsivity. This phenomenon will be referred to as “oculomotor impulsivity” in this study. Additionally, according to the literature from above, short-term temporal memory and inhibitory control could potentially be altered and disrupt the FP effect in depressive patients. Thus, secondly, we further examined whether short-term temporal memory and inhibitory control affects temporal preparation in participants diagnosed with depression.

## Materials and Methods

### Ethical Approval and Informed Consent

This study was approved by the Joint Institutional Review Board, Taipei Medical University, Taipei City, Taiwan (N201603080). Methods were carried out in accordance with relevant guidelines and regulations. All participants were informed about the purpose of the study and procedures before being asked to give informed consent. Written informed consent was obtained from all participants prior to their participation in this study.

### Patients

Twenty-nine patients diagnosed with MDD (24 females; 38.4 ± 2.5 years old, *n* = 29) were recruited by the Department of Psychiatry at Taipei Medical University Shuang-Ho Hospital, located in New Taipei City, Taiwan. The MINI-international neuropsychiatric interview (Sheehan et al., 1998), was used to confirm the diagnosis of current major depressive episode, to detect suicidal risk, and to exclude patients with psychotic symptoms and any comorbid mental disorder or substance use disorder according to the Diagnostic and Statistical Manual of Mental Disorders, Fifth Edition. Patients with poor visual acuity and comorbid medical conditions including neurological disorders (e.g., stroke, seizure, traumatic brain injury, post-brain surgery), brain implants (neurostimulators), cardiac pacemakers, or pregnant were also excluded. The Beck Depression Inventory (BDI-II; Beck et al., 1996), and the Generalized Anxiety Disorder 7 (GAD-7; Spitzer et al., 2006) were administered to evaluate the severity of depression and anxiety. BDI-II score for patients in this study was 30.1 ± 13.4 and depressive symptoms duration was 9.2 ± 10.2 years. In addition, the BIS-11 (Patton et al., 1995) was used to quantify Impulsive level on each individual. All patients but seven were on medication at the time of testing (see Table 1).

**TABLE 1:** Summary of drug treatments received by patients.

**Patient**	**1**	**2**	**3**	**4**	**5**	**6**
p01	SNRI	Non-BDZ	BDZ	ATA	BDZ	
p03	MRA	Non-BDZ				
p04	SSRI	BDZ				
p05	BDZ	Non-BDZ	NDRI			
p06	**NIL**					
p07	ATA	SSRI				
p08	NDRI					
p09	MRA					
p11	SSRI	ATA				
p12	SSRI	ATA	NDRI			
p13	SNRI	ATA				
p14	SSRI					
p15	SNRI	Non-BDZ	BDZ			
p17	**NIL**					
p18	SSRI					
p19	**NIL**					
p20	**NIL**					
p21	SNRI	Non-BDZ	Non-BDZ	SARI	ATA	ATA
p25	SSRI					
p26	SSRI	BDZ	BDZ			
p28	SNRI	ATA	BDZ	BDZ	SARI	BDZ
p29	SSRI	BDZ	ATA			
p30	Non-BDZ	BDZ				
p31	NDRI					
p32	**NIL**					
p34	**NIL**					
p35	SNRI	BDZ	Non-BDZ	ATA		
p37	SNRI	ATA				
p41	**NIL**					

*Each line represents one patient. Columns show treatments received by each patient (1–6). ATA, atypical antipsychotic; BDZ, benzodiazepine; SNRI, serotonin–norepinephrine reuptake inhibitor; MRA, melatonin receptor agonist; NDRI, norepinephrine–dopamine reuptake inhibitor; **NIL**, no pharmacological treatment; Non-BDZ, non-benzodiazepine hypnotic; SARI, serotonin antagonist and reuptake inhibitor; SSRI, selective serotonin reuptake inhibitor.*

### Controls

Twenty-nine healthy control participants (27 females, 37.7 ± 2.4 years old) without any current or history of neurological or psychiatric disorder, or use of psychotropic medication, were recruited from the community. They were matched for age and gender, except for two healthy control participants, whose gender did not match the patients.

### Experimental Design and Statistical Analysis

**FIGURE 1:**
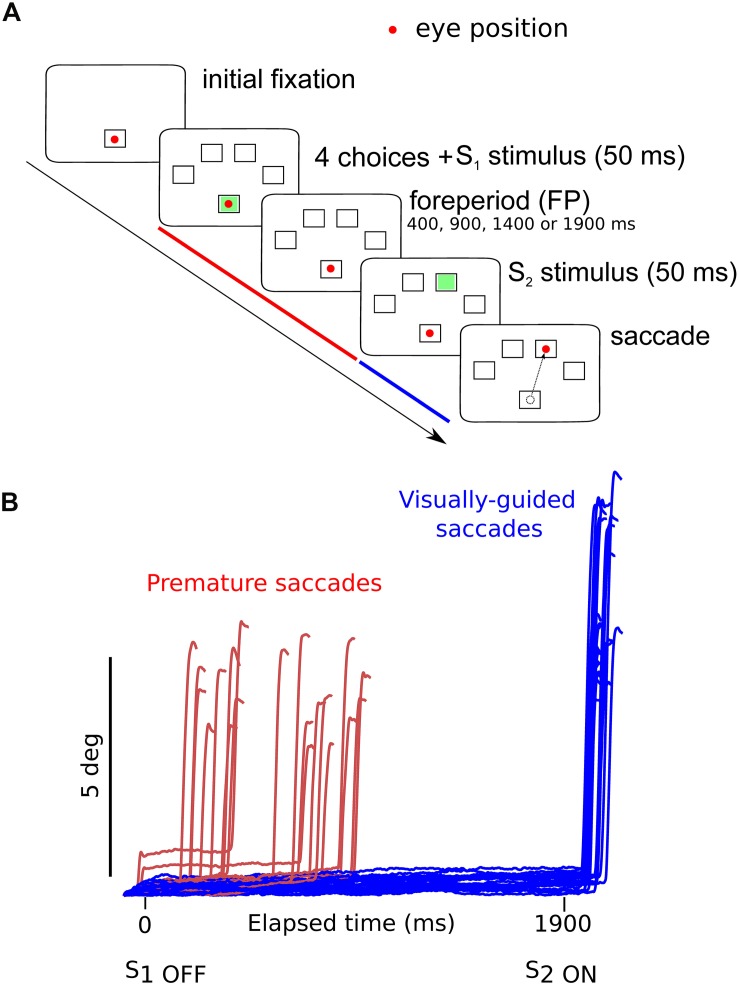
**(A)** Oculomotor version of the 4-CSRTT task. Schematic representation of the visual display in front of subjects. See text for details. **(B)** Example of premature (*red traces*) and visually guided (*blue traces*) saccades during a 1900-ms foreperiod in a control subject (subject #9). Time zero on the *X*-axis indicates extinction of the warning stimulus (S_1__OFF_). At the end of the FP, the imperative stimulus appeared (S_2__ON_). Note that most premature saccades tended to cluster during the early part of the FP. The period during which premature saccades were recorded is represented in *red* in panel **(A)**. The period during which visually guided saccades were recorded is represented in *blue* in panel **(A)**.

Subjects were facing an LCD screen which presented stimuli at a refresh rate of 60 Hz. An EyeLink 1000 infrared eye tracking system (SR Research, Mississauga, ON, Canada) was used to record eye movements at 1 KHz. Saccade initiation was measured using the algorithm provided by SR Research. This algorithm uses a saccadic velocity threshold of 30°/s, a saccade acceleration threshold 8000°/s^2^ and a saccade motion threshold of 0.15°. Stimulus display and oculomotor data collection were synchronized on a frame-by-frame basis using Experimental Builder (SR Research, Mississauga, ON, Canada). Figure 1A depicts the time line of stimuli presentation on the screen facing the subject. Each trial started with an initial fixation period of a small empty box (1.4 × 1.4°) appearing on the screen for a random duration (850 ± 100 ms; Figure 1A). At the end of this period four additional empty square “boxes” appeared on the screen at an eccentricity of 8° together with a warning stimulus S_1_ that was briefly presented in the central box for 50 ms. Extinction of the S1 stimulus indicated to subjects the beginning of the foreperiod (FP). Subjects were required to fixate on the central box until a target was briefly and randomly presented for 50 ms, in one of the four eccentric boxes (imperative stimulus S_2_). The background of the screen was always black and stimuli were white (boxes) or green (S_1_ and S_2_ stimuli). One of four different FP durations (400, 900, 1400, and 1900 ms) was chosen randomly, each with the same probability (Probability = 0.25). Subjects were required to wait until targets appeared in the eccentric box before making a saccade (black arrowhead on Figure 1A). Saccadic latency (reaction time) was defined as the time elapsed between the appearance of the eccentric target and movement onset. Saccades that occurred during the foreperiod (period indicated with a *red line* on Figure 1A) are here referred to as “premature saccades” (*red traces* on Figure 1B). The propensity to initiate premature saccades will be referred to as “oculomotor impulsivity.” Saccades that occurred after the appearance of the eccentric target (period indicated with a *blue line* on Figure 1A) are here referred to as “visually guided saccades” (*blue* traces on Figure 1B). The amplitude of visually guided saccades had to be >5° This criterion was rendered necessary in order to eliminate dysmetric primary saccades between the S_1_ and S_2_ stimuli. Dysmetric primary saccades were more frequent in MDD and were not included in the analyses presented here (see Sweeney et al., 1998).

In order to suppress express saccades, saccadic latency had to be longer than 100 ms. In order to avoid long latency saccades likely initiated in a decreased state of alertness or motivation, saccade latency had to be less than 600 ms. Altogether, these conservative criteria eliminated 5% of the total number of saccades in controls (5377/5683 saccades) and 13% in MDD patients (4074/4710 saccades).

At the end of each trial, boxes were removed and there was a variable inter-trial-interval of between 2000 and 2500 ms. A block contained 120 trials and each subject performed two blocks for a total of 240 trials. This paradigm is an oculomotor version of the well-known four choices serial reaction time task (i.e., 4-CSRTT) used in humans (Voon et al., 2014).

A linear mixed model approach (LMM) was used to analyze eye movement data. In all analyses, subject identity was used as a random factor to account for the influence of uncontrolled, between-subject variability. To compare the percentage of premature responses, a single average value was computed for each subject before applying the LMM analysis and testing for fixed effects. In order to analyze saccadic latencies, we used a *repeated-measures* LMM. In this analysis, each saccadic latency measured for each subject was a data point. Indeed, the LMM approach does not require a preliminary averaging of data for each subject and condition. Therefore, it avoids information loss due to data averaging (see West et al., 2015; Boisgontier and Cheval, 2016) and is more selective to test experimental effects and interactions (Baayen et al., 2008). The LMM method is also more robust to normality violations and missing data (e.g., no saccade triggered) than standard ANOVA or ANCOVA.

In summary, LMM provides unbiased analysis of balanced and unbalanced repeated-measures data, detection of within-subject effects (fixed effects), and individual subject effects (random effects), thereby making the best use of all available data (Laird and Ware, 1982).

The number of degrees of freedom (df) was estimated using the Satterthwaite algorithm calculated by the MIXED algorithm in SPSS 25 (SPSS Inc., Chicago, IL, United States). With this algorithm, the number of df could be fractional. Significance of observed effects was tested using the F-statistics. The significance threshold α for all analysis was 0.05.

Results are presented as mean ± standard error of the mean, unless otherwise specified. Because saccadic reaction time distributions are often non-normal, we used a logarithmic transform of saccadic RT for statistics. For presentation purposes, however, untransformed saccadic latencies in milliseconds are presented on figures.

A preliminary version of this paper was posted on bioRxiv by Hsu et al. (2019).

## Results

### Demographics and Clinical Characteristics

Average age of control subjects was 37.7 ± 2.4 (*n* = 29) and 38.4 ± 2.5 (*n* = 29) for patients. Age did not differ between groups (*F*[1,56] = 0.048; *p* = 0.827; *n* = 29). Average scores of the Beck’s Depression Inventory-II were 7.6 ± 1.4 in controls (*n* = 25/29; 4 untested controls) and 30.1 ± 13.4 in patients (*n* = 22/29; seven untested patients). These group scores were statistically different (χ^2^ = 20.455; *p* < 0.001) confirming the diagnosis of MDD. Average total scores of the BIS-11 were 64.6 ± 1.2 in controls (*n* = 29/29) and 71.3 ± 2.0 in patients (*n* = 27/29). These between-group scores differed significantly, (χ^2^ = 4.577; *p* = 0.032) with patients scoring higher than controls.

### Premature Saccades

**FIGURE 2:**
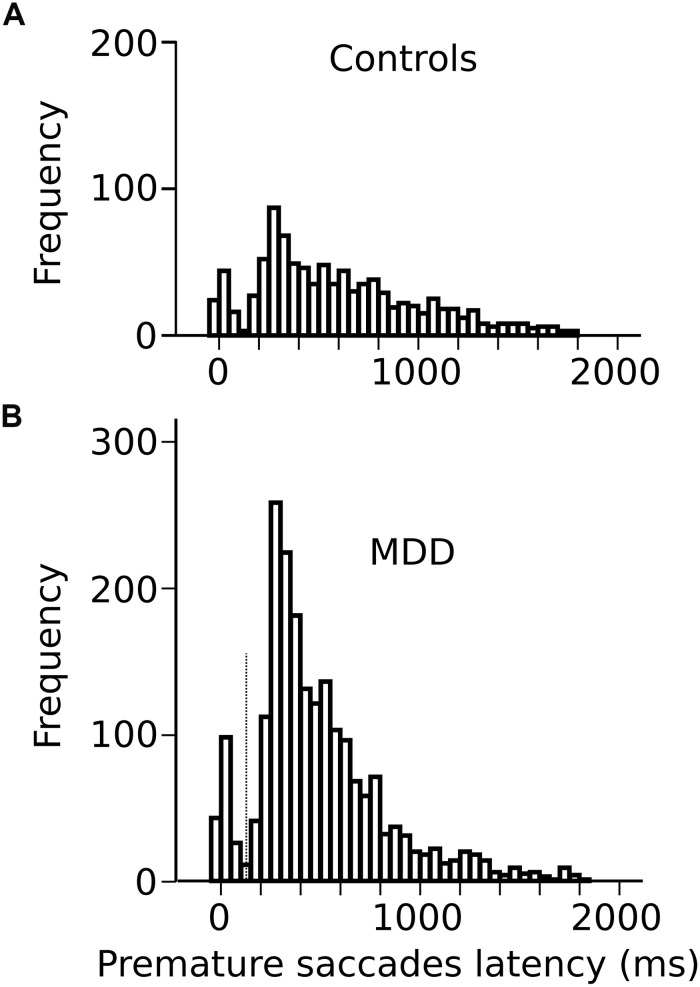
**(A)** Latency distribution of all premature saccades in controls pooled together. **(B)** Latency distribution of all premature saccades in MDD patients. Time zero on the *X*-axis represents disappearance of the S_1_ stimulus.

In the oculomotor version of the 4-CSRTT task, subjects were instructed to keep looking at the S_1_ stimulus in the central fixation box and wait for the appearance of the eccentric S_2_ stimulus before initiating a visually guided saccade. However, saccades were often initiated before the appearance of the S_2_ stimulus (see *red* traces on Figure 1B). These movements will be referred to as “premature” saccades. Figure 2 shows the latency distribution of premature saccades for all controls (Figure 2A) and patients (Figure 2B). Most premature saccades occurred approximately 200 ms after the offset of the warning stimulus. The percentage of premature saccades was measured and served as an index of inhibition. We found that the percentage of premature saccades was more than twice as high in patients than in controls (in controls: 14 ± 2%, *n* = 29 subjects, 937 premature saccades/6620 trials; in patients: 31 ± 5%, 2064 premature saccades/6776 trials) and there was a significant main effect of subject group on this percentage (*F*[1,56] = 8.4; *p* = 0.005). Premature saccade latency was longer in controls (598 ± 13 ms; *n* = 937) than in MDD patients (491 ± 7 ms; *n* = 2066; *F*[1,2926] = 13.410; *p* < 0.001) supporting the hypothesis of increased oculomotor impulsivity in patients. Next, we investigated whether there was a correlation between trait impulsivity as estimated with the BIS-11 questionnaire and oculomotor impulsivity. However, we found no correlation between the percentage of premature saccades and the BIS-11 score (in MDD patients: *F*[1,20] = 0.34; *p* = 0.856; *n* = 22; seven patients not tested; in controls: *F*[1,23] = 0.216; *p* = 0.647; *n* = 25; four subjects not tested) suggesting that these measures reflect different facets of impulsivity.

### Influence of Temporal Preparation on Visually Guided Saccades

**FIGURE 3:**
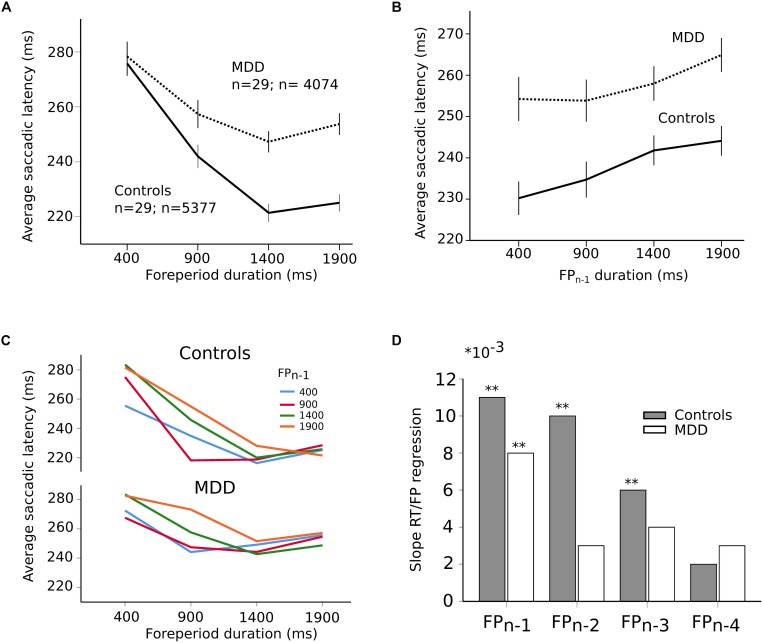
**(A)** RT-FP function in controls (*continuous* line; *n* = 29 subjects; *n* = 5377 saccades) and MDD patients (*dotted* line; *n* = 29 subjects; *n* = 4074 saccades). Average values of saccadic latency (in milliseconds) and 95% confidence interval. Note the shallower slope of the function between 400 and 1400 ms FP durations. **(B)** Relationship between previous foreperiod duration (FP*_n_*_–__1_) and saccadic latency in controls (*continuous* line) and MDD patients (*dotted* line). Same data set as on panel **(A)**. **(C)** Asymmetry of the foreperiod effect. Colors shows the duration of the previous FP (FP*_n_*_–__1_). Same data as in panel **(A)** but with a classification of saccadic latency according to previous FP. The influence of FP*_n_*_–__1_ is larger for short FP durations. **(D)** Graphical representation of the slope of the linear relationships between previous FPs and saccadic latency for an increasing number of trials back into the past (1–4) in controls (*dark* bars) and MDD patients (*open* bars). Same data set as on panel **(A)**. The symbol ^∗^ is used to represent multiplication; ^∗∗^ indicates *P* < 0.01.

Most saccades were visually guided, as expected, in accord with the instructions provided to subjects (*blue traces* on Figure 1B; 76% visually guided saccades, 9451/12452 saccades all subjects included; average latency 239 ± 1 ms, *n* = 5377 saccades; MDD: 259 ± 1 ms, *n* = 4074 saccades). Figure 3A shows the relationship between saccadic latency and foreperiod duration in the two groups of subjects. This relationship will be referred to as the RT-FP function. It can be observed that saccadic latencies were similar between groups, if current FP duration was 400 ms. But there was an increasing difference between groups for FP duration longer than 400 ms. We found a statistically significant main effect of FP duration (*F*[3,9394.324] = 274.374; *p* < 0.001) on saccadic latency, but no significant main effect of group on average saccadic latency (*F*[1,55.082] = 0.472; *p* = 0.495). The interaction between FP duration and subject group was significant (*F*[3,9394.324] = 22.715; *p* < 0.001). These results show that *average* reaction time was statistically similar in controls and MDD subjects but that temporal preparation was reduced in the latter group.

In order to better understand the origin of the different RT-FP functions between groups, we analyzed the influence of previous FP duration (FP*_n_*_–__1_) on saccadic latency during the current FP. For instance, if current FP duration was 400 ms and FP*_n_*_–__1_ was 400 ms as well, saccadic latency could be shorter than if the same FP was preceded by FP*_n_*_–__1_ of 1900 ms (see Figure 3B). Statistically, we found that there was a significant main effect of FP*_n_*_–__1_ duration (*F*[3,9314.030] = 22.841; *p* < 0.001) on saccadic latency, but not subject group (*F*[1,55.229] = 1.077; *p* = 0.304). The interaction between FP*_n_*_–__1_ duration and subject group was not significant (*F*[3,9314.030] = 1.633; *p* = 0.179). These results suggest that the FP*_n_*_–__1_ effect was present and similar in both controls and patients. Therefore, the reduced FP effect that we found could not be due to a reduced influence of FP*_n_*_–__1_ duration held in short-term memory. This conclusion was reinforced by the analysis presented on Figure 3C that shows the influence of FP*_n_*_–__1_ as a function of FP duration in controls and MDD patients. The influence of FP*_n_*_–__1_ was stronger for short FP durations than longer ones. This effect is often referred to as the “asymmetry” of the sequential effect (Los et al., 2017). This asymmetry was present in both groups and was statistically similar (no significant interaction between FP, FP*_n_*_–__1_ and subject group: *F*[9,9290.307] = 1.38; *p* = 0.190]). However, more than one step back into the past could still play a significant role in the timing of eye movements and explain the different RT-FP functions between groups. Therefore, we investigated whether FP*_n_*_–__1_, FP*_n_*_–__2_, FP*_n_*_–__3_, FP*_n_*_–__4_ could also play a significant role in determining saccadic latency during the current FP*_n_* in both groups. We found that FP*_n_*_–__1_, FP*_n_*_–__2_, FP*_n_*_–__3_, and FP*_n_*_–__4_ played a significant role in determining saccadic latency in controls (FP*_n_*_–__1_, *F*[3,4935.335] = 16.090; *p* < 0.001; FP*_n_*_–__2_, *F*[3,4935.316] = 9.382; *p* < 0.001; FP*_n_*_–__3_, *F*[3,4935.283] = 4.126; *p* = 0.006; FP*_n_*_–__4_, *F*[3,4935.237] = 3.562; *p* = 0.014). But only FP*_n_*_–__1_ played a significant role on saccadic latency in MDD patients (FP*_n_*_–__1_, *F*[3,3654.868] = 6.270; *p* < 0.001). Therefore, the reduced FP effect found in patients could be attributed to a different processing of past FP durations, with a faster decay of the memory trace of previous FPs in MDD patients. This is further illustrated on Figure 3D, which shows the value of the slope of a multiple linear regression analysis between previous FPs and saccadic latency. Star symbols indicate when slopes were significantly different from zero. This figure shows a faster extinction of the influence of previous FPs on movement latency in MDD patients than in controls.

Analyses presented above suggest that short-term temporal memory of previous FPs affected saccadic latency in MDD patients differently than in controls. Temporal preparation, however, could also be guided by a sense of elapsed time during the current FP. Therefore, we applied a hierarchical linear regression analysis in both groups using two different models. In controls, the first model contained FP*_n_*_–__1_, FP*_n_*_–__2_, FP*_n_*_–__3_, and FP*_n_*_–__4_ as independent factors. The second model contained FP*_n_*_–__1_, FP*_n_*_–__2_, FP*_n_*_–__3_, FP*_n_*_–__4_, and FP*_n_* as independent factors and will be referred to as “model 2.” The aim was to determine the contribution of both short-term temporal memory (FP*_n_*_–_*_*x*_*) and current foreperiod (FP*_n_*) in movement latency. In controls, we found that both model 1 (*F*[4,5213] = 17.269; *p* < 0.001) and model 2 (*F*[5,5212] = 89.276; *p* < 0.001) provided a significant explanation of observed variance. The coefficient of determination for model 1 was *r*^2^ = 0.013 (*p* < 0.001); however, the coefficient of determination for model 2 was higher with *r*^2^ = 0.079. Therefore, *r*^2^ variation due to adding current FP*_n_* in the model was approximately 7% (0.066) and the *F*-value variation related to this addition was significant (*F*[1,5212] = 372.384; *p* < 0.001). In patients, we found that both model 1 (*F*[1,4038] = 12.559; *p* < 0.001) and model 2 (*F*[2,4037] = 38.633; *p* < 0.001) also provided a significant explanation of the variance observed. The coefficient of determination for model 1 was weak *r*^2^ = 0.003 but significant (*p* < 0.001). But the coefficient of determination for model 2 was higher with *r*^2^ = 0.018 and the *r*^2^ variation due to adding FP*_n_* in the model was approximately 2% (0.016). *F*-value variation related to the addition of FP*_n_* in the model was significant (*F*[1,4037] = 64.511; *p* < 0.001). In summary, the FP effect was observed in both groups but in MDD patients memory of previous FPs declined faster, given that only FP*_n_*_–__1_ played a significant role on saccadic latency. Using a hierarchical model analysis, we showed that the model including previous FPs and current FP*_n_* explained a larger proportion of the variance of saccadic latency. Influence of the current FP was present in both controls and patients but its influence was weaker in the latter group. Therefore, the reduced influence of current FP on saccadic latency co-occurred with a reduced short-term temporal memory in MDD patients.

### Was Short-Term Memory in the Spatial Domain Similarly Affected in MDD Patients?

**FIGURE 4:**
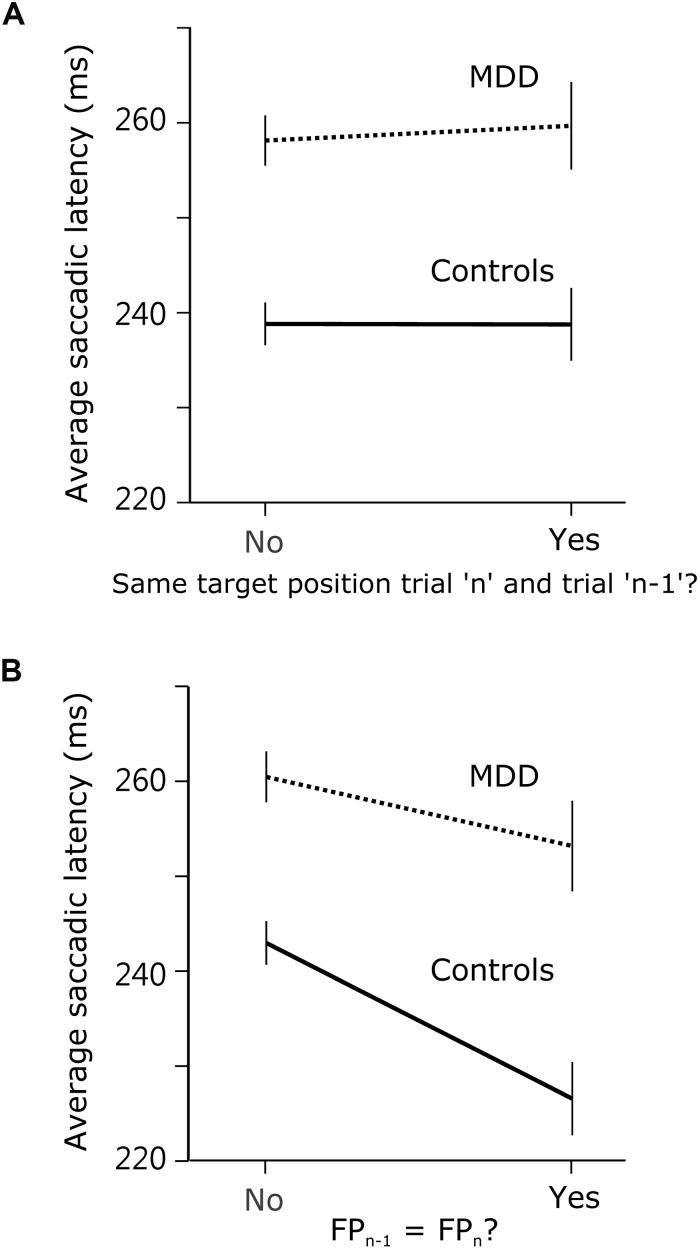
Comparison of spatial and temporal short-term temporal memory. **(A)** Saccadic latency in MDD and control subjects when the visual target was at the same position during trial*_n_* as during trial*_n_*_–__1_, or not. **(B)** Saccadic latency when FP duration was the same during trial*_n_* as during trial*_n_*_–__1_, or not.

If the answer to this question were positive, then observed effects were not specific to temporal preparation. Instead, they might reflect a more general short-term memory impairment in patients. In order to determine whether there was a significant effect of previous target *position*, we compared saccadic latency when the target appeared at the same or at a different spatial location during the previous trial. We hypothesized that there could be a response facilitation by repetition in the temporal domain only, but not in the spatial domain. Indeed, we found no evidence of a significant interaction effect between subject group and previous target *location* on saccadic latency (*F*[1,9318.688] = 0.081; *p* = 0.776; see Figure 4A). We applied the same approach to temporal preparation by comparing saccadic reaction time when FP*_n_* and FP*_n_*_–__1_ were the same or different. In the temporal domain, there was a significant interaction between subject group and FP duration on saccadic latency (same or different; *F*[1,8907.674] = 4.077; *p* = 0.043; see Figure 4B). These results show that *temporal* short-term memory was selectively affected in patients, but the same was not true for *spatial* short-term memory of target location.

### Influence of Oculomotor Impulsivity on Temporal Preparation

**FIGURE 5:**
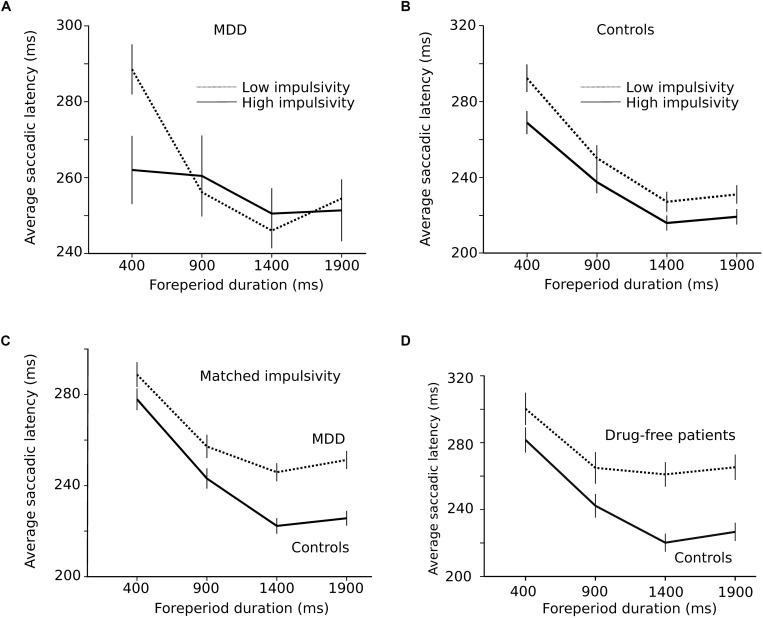
**(A)** Influence of impulsivity on the RT-FP function in MDD patients (low impulsivity; *n* = 15 subjects; *n* = 2864 saccades; high impulsivity group: *n* = 14 subjects, *n* = 1210 saccades). **(B)** Same relationship in controls (low impulsivity; *n* = 15 subjects; *n* = 2864 saccades; high impulsivity group: *n* = 14 subjects, *n* = 1210 saccades). **(C)** RT-FP function for patients (*n* = 19, nine subjects removed; *n* = 3546 saccades) and controls (*n* = 26, two subjects removed; *n* = 4972 saccades) that were matched for oculomotor impulsivity. **(D)** RT-FP function in seven untreated patients (*n* = 1305 saccades) and 7 age-matched controls (*n* = 1530 saccades).

As hypothesized in the *Introduction* section, the shape of the RT-FP function could be influenced by oculomotor impulsivity. Therefore, we compared the RT-FP functions in low and high oculomotor impulsivity patients and controls. Patients were categorized into two groups, using the median of the percentage of premature saccades distribution in this group (median = 21%). The low impulsivity MDD group produced less than 21% premature responses (*n* = 15 subjects; *n* = 2864 saccades); the high impulsivity MDD group, more than 21% (*n* = 14 subjects; *n* = 1210 saccades). We re-examined the FP effect within these two groups. Figure 5A shows that the asymmetric FP effect was present in the low impulsivity group, but it was considerably altered in the high impulsivity group. Accordingly, a significant interaction between impulsivity group and FP duration on saccadic latency was found (*F*[3,4048.361] = 4.655; *p* = 0.003). In high oculomotor impulsivity patients, saccadic latencies for the 400 ms duration FP were reduced by approximately 30 ms. Figure 5B shows the same analysis applied to control subjects (median percentage of premature saccades: median = 10%). The RT-FP function in high (*n* = 15 subjects; *n* = 2313 saccades) and low impulsivity (*n* = 14 subjects; *n* = 3064 saccades) control subjects also significantly differed (significant interaction between impulsivity group and FP duration on saccadic latency; *F*[3,5342.181] = 4.125; *p* = 0.006), but to a lesser extent. Indeed, as already mentioned, oculomotor impulsivity was lower in this group; therefore, its influence on the RT-FP relationship was less. In summary, impulsivity increased temporal preparation to the temporally proximal target in both controls and patients, with a more pronounced effect in the latter group. It could be hypothesized that the shallower RT-FP relationship in impulsive patients could be partly attributed to a reduced asymmetry of the sequential short-term memory effect. However, we found no interaction between FP*_n_*, FP*_n_*_–__1_ and subject group (*F*[9,4008] = 0.691; *p* = 0.717; see Supplementary Figure 1).

The perception of elapsed time, however, could also be modified by depression, independent of impulsivity. In order to test this hypothesis, patients and controls were matched for oculomotor impulsivity. If the shape of the RT-FP relationship was different for the same oculomotor impulsivity, then depression *per se* is affecting temporal preparation. In order to obtain matched oculomotor impulsivity groups, we removed patients and subjects producing more than 30% premature responses and identified two overlapping groups — controls (*n* = 26, 2 subjects removed; *n* = 4972 saccades) and depressed patients (*n* = 19, 9 subjects removed; *n* = 3546 saccades) producing 12 and 14% of premature responses, respectively. Figure 5C shows that the RT-FP functions were different, nevertheless, in patients and controls (significant interaction; *F*[3,8467.292] = 13.082; *p* < 0.001) with a reduced FP effect in patients. Therefore, the shape of the RT-FP function could be altered by both oculomotor impulsivity and depression.

One additional factor that could influence temporal preparation is drug treatments administered to MDD patients. Most subjects (22/29) received several drug treatments that could have influenced temporal preparation (see Table 1). Due to ethical considerations, it would not have been acceptable to ask patients to suspend their treatment for research purposes. But seven subjects were investigated before the start of the treatment and temporal preparation could be estimated (*n* = 1305 saccades). Figure 5D shows a strong alteration of the RT-FP function for drug-free patients, compared to age-matched controls (*n* = 1530 saccades). This subset of seven, untreated subjects presented a similar alteration of the RT-FP function as was found when all subjects were pooled together (see Figure 3A), and a significant interaction between group and FP duration on saccade latency was found (*F*[3,2815.141] = 10.639; *p* < 0.001). This result suggests that temporal preparation alteration in depression is a robust observation, and that it is not caused by drug treatment. However, we found no correlation between the BDI score and the slope of the RT-FP relationship (Pearson correlation, −0.032, *p* = 0.88, *n* = 22).

### Influence of Trait Impulsivity on Temporal Preparation

In the present study, the percentage of premature responses was used to evaluate oculomotor impulsivity. The percentage of premature responses was not correlated with the BIS-11 score (see above). However, could the BIS-11 score predict the shape of the RT-FP function? In order to answer this question, we divided MDD patients into a high and low impulsivity groups based on the median of the total BIS-11 score. The same LMM procedure was applied to test whether the RT-FP function was different between groups. However, we found that there was no main effect of trait impulsivity on saccadic RT (*F*[1,24.250] = 0.001; *p* = 0.973). Moreover, no significant interaction was found between trait impulsivity and FP duration (*F*[3,3777.549] = 2.235; *p* = 0.082). In conclusion, temporal preparation was not significantly altered by trait impulsivity.

## Discussion

The aim of this study was to determine how temporal preparation and short-term temporal memory were affected in MDD as well as the influence of the lack of inhibitory control (or impulsivity). In general, we found that temporal preparation was reduced in MDD compared with age-matched healthy controls. We suggest that psychomotor retardation could not fully explain observed results. Indeed, our analysis revealed that *average* reaction time to visual targets was not statistically different between groups. We found that temporal preparation depended on both the duration of the current FP and short-term memory of previous FPs. In MDD, the influence of current FP duration was reduced and the decay of short-term temporal memory was faster. Indeed, the influence of previous FPs was 3–4 trials deep in controls, but only one trial deep in depressed patients. A significant reduction of the FP effect was found in seven MDD patients who were not undergoing pharmacological treatment; this finding suggests that abnormal temporal preparation occurs independently of therapeutic drugs. In addition, we found that lack of inhibition also influenced temporal preparation. The RT-FP function was statistically flat in high oculomotor impulsivity patients.

### Premature Saccades and Oculomotor Impulsivity

Premature saccades reflected the lack of inhibitory control during the foreperiod. Inhibitory control plays a crucial role while waiting for an expected event (Logan et al., 1997) and motor responses occurring before the appearance of the imperative S_2_ stimulus must be actively suppressed. We observed that premature saccades clustered between 200 and 400 ms after S_1_ offset. We suggest that two hypotheses could explain this observation. First, there could be an increased expectancy of S_2_ appearance after a short FP. This increased expectancy could be caused by a more salient representation of the short FP in memory. Second, response inhibition might be reduced at the beginning of the FP. But the observation that short-term temporal memory was reduced in MDD patients does not support the first hypothesis. Indeed, a reduced memory span and an increased saliency of memory traces of short FPs are inconsistent. On the other hand, the second hypothesis is supported by observations made in humans and rodents. It has been suggested that the frontal and prefrontal cortices could exert inhibitory control over behavior, cognition and emotions although there is not yet a consensus about the networks involved in the different domains of inhibition (Jahanshahi and Rothwell, 2017). Interestingly, Narayanan et al. (2006) have shown that in rat reversible inactivation of the dorsomedial prefrontal cortex caused an increase in premature responses and an accelerated reaction time after a short foreperiod, just as was observed in the present study. Therefore, premature saccades might reveal a lack of top-down inhibitory control exerted by the prefrontal cortex; this would be particularly important at the beginning of the FP. Later, during the FP, an excitatory drive could prepare movement initiation when the imperative stimulus occurs. The inhibitory control normally opposes this excitatory drive. But if the inhibitory drive were reduced then the excitatory drive could trigger either a premature response during the FP or a short latency saccade to the imperative visual target during a 400 ms FP. An increased occurrence of premature responses is often observed in psychiatric disorders and addictions where impulsivity is increased (Dalley and Robbins, 2017; Paasche et al., 2018). Among psychiatric diseases, MDD seems to be a special case. It is usually associated with heightened inhibitory control and reduced motor activity (American Psychiatric Association, 2013). But increased impulsivity has also been reported (Corruble et al., 2003) and top-down inhibitory control is likely to be affected by MDD (Palmwood et al., 2017). This hypothetical reduction of top-down inhibitory control in MDD could be related to reduced serotoninergic neurotransmission. Indeed, it has been shown in humans that a dietary tryptophan depletion procedure that reduces serotonin neurotransmission causes waiting impulsivity (Dalley and Roiser, 2012; Worbe et al., 2014). Therefore, increased oculomotor impulsivity could be a consequence of altered serotoninergic transmission in depressed patients (Coppen, 1967; see Yohn et al., 2017). The saccadic system is a high gain system constantly kept under strong inhibitory control in order to avoid unwanted eye movements (Missal and Keller, 2002; Otero-Millan et al., 2018) and this delicate balance could be easily perturbed by reduced top-down inhibitory control (see review in Pouget et al., 2017). Additionally, increased impulsivity could also be related to therapeutic drugs taken by subjects. Indeed, the RT-FP function of untreated subjects was similar to the one observed in low-impulsivity patients in general. Measuring the percentage of premature saccades could be a clinical tool to estimate the influence of pharmacological treatment on impulsive behavior.

The absence of correlation between the percentage of premature saccades and *trait* impulsivity (BIS-11 total score) supports the hypothesis that impulsivity is a complex construct with different aspects and several different neurotransmitters involved (Evenden, 1999; Dalley and Robbins, 2017). Premature responses characterize the motor side of impulsivity but does not reflect higher order cognitive aspects of this phenomenon.

### The Origin of Temporal Preparation

Trace conditioning model suggests that the shape of the RT-FP function could be explained by a single learning mechanism based on previously experienced FPs (Los et al., 2014). In this model, inhibition during FP*_n_*_–__1_ causes a longer RT during the current FP. This explains the asymmetry of the FP effect. Therefore, less inhibitory control (oculomotor impulsivity) should be associated with a reduced influence of short-term temporal memory and a shallower slope of the RT-FP function in MDD. Although the slope of the RT-FP function was reduced in impulsive patients, there was still a significant influence of FP*_n_*_–__1_. We could not validate this prediction of the MTP model in our data set. However, an updated model from the same group, the multiple tract theory of temporal preparation (abbreviated as “MTP”; Los et al., 2017) suggested that the shape of the entire RT – FP function reflects accumulated inhibition across all previous trials (stored in memory traces). That is, the complete area below the RT – FP function is an expression of accumulated inhibition. The asymmetry of the sequential effect is a reflection of inhibition according to MTP, where recent memory traces have a higher weight. Our findings also support that the FP effect has multiple origins (Vallesi and Shallice, 2007; Vallesi et al., 2007a, b) both affected in MDD.

When controls and patients were matched for impulsivity, temporal preparation was nevertheless reduced in the latter group. Therefore, impulsivity alone cannot explain observed results and depression by itself reduced temporal preparation. We suggest that depression could reduce the influence of elapsed time on movement latency for long FPs.

Using the exquisite temporal sensitivity of the oculomotor system we have shown that the *implicit* processing of time is altered in MDD patients. This implicit processing of time probably involves early neuronal activity in the frontal cortex. It has been shown by Los and Heslenfeld (2005) that the effects of previous FPs were paralleled by similar effects on the fronto-central contingent negative variation (CNV). We suggest that this modulation of the CNV by sequence effects (or short-term temporal memory) should be reduced in MDD.

### Implicit Timing, Time Perception, and Awareness

Based on questionnaires, depressed patients report often a “slowing down” of subjective time (Blewett, 1992; Ratcliffe, 2012; see review in Droit-Volet, 2013). This desynchronization between the subjective experience of time and physical time seems to be a characteristic of depressive states without being systematically studied and compared with other cognitive functions. How temporal preparation, explicit timing and awareness of time relate to each other is unknown. Although temporal preparation and time awareness could be considered as very different phenomena they could rest on overlapping neurophysiological mechanisms. We suggest that alteration of these mechanisms could underlie the abnormal temporal organization of thought and behavior often reported in this disease.

## Data Availability Statement

The datasets generated for this study are available on request to the corresponding author.

## ETHICS STATEMENT

The studies involving human participants were reviewed and approved by the Joint Institutional Review Board, Taipei Medical University, Taipei City, Taiwan (N201603080). The patients/participants provided their written informed consent to participate in this study.

## Author Contributions

MM and T-YH designed the research and wrote the manuscript. T-YH performed the research. H-CL contributed the unpublished reagents and analytic tools. TL wrote the manuscript.

## Conflict of Interest

The authors declare that the research was conducted in the absence of any commercial or financial relationships that could be construed as a potential conflict of interest.
