# Supplementary figures and images for: Temporal Preparation, Impulsivity and Short-Term Memory in Depression
Source: Front Behav Neurosci. 2019 Nov 22;13:258. doi: 10.3389/fnbeh.2019.00258 (PMC6882746; doi:10.3389/fnbeh.2019.00258)

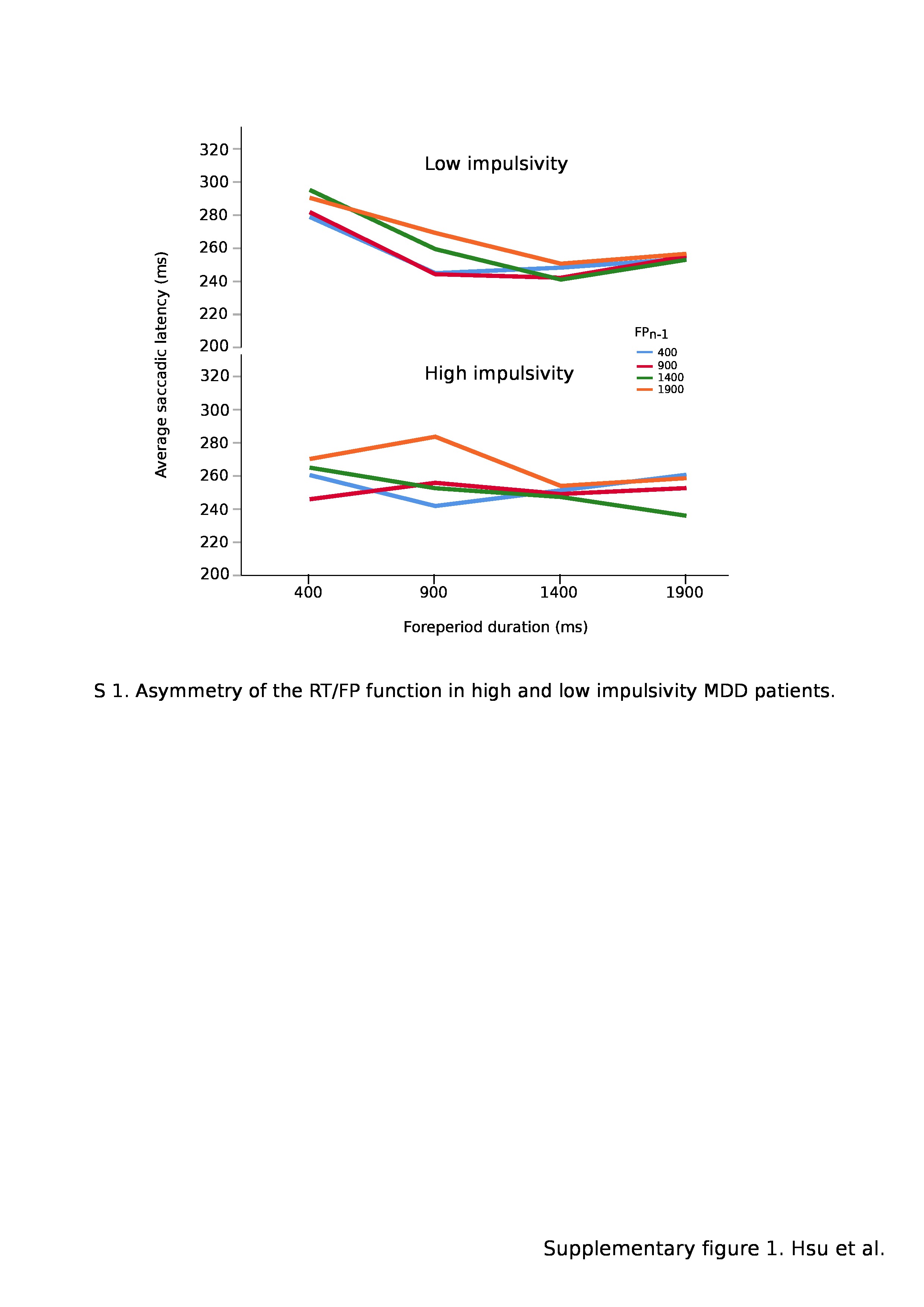

Supplement: Supplementary file 1 [file Image_1.JPEG]
